# The Role of IL-35 in the Pathophysiological Processes of Liver Disease

**DOI:** 10.3389/fphar.2020.569575

**Published:** 2021-01-21

**Authors:** Shuang Hu, Pan-pan Lian, Ying Hu, Xing-yu Zhu, Shao-wei Jiang, Qiang Ma, Liang-yun Li, Jun-fa Yang, Li Yang, Hai-yue Guo, Hong Zhou, Chen-chen Yang, Xiao-ming Meng, Jun Li, Hai-wen Li, Tao Xu, Huan Zhou

**Affiliations:** ^1^ Inflammation and Immune Mediated Diseases Laboratory of Anhui Province, Anhui Institute of Innovative Drugs, School of Pharmacy, Anhui Medical University, Hefei, China; ^2^ Institute for Liver Diseases of Anhui Medical University, Hefei, China; ^3^ School of Pharmacy, NanJing University, NanJing, China; ^4^ National Drug Clinical Trial Institution, The First Affiliated Hospital of Bengbu Medical College, Bengbu, China; ^5^ The First Affiliated Hospital of Anhui Medical University, Hefei, China; ^6^ Second Affiliated Hospital of Anhui Medical University, Hefei, China; ^7^ The Third Affiliated Hospital of Anhui Medical University, The First Affiliated Hospital of Zhengzhou University, Zhengzhou, China

**Keywords:** Interleukin-35, liver diseases, lipid accumulation, cellular proliferation, hepatocellular carcinoma

## Abstract

It is known that liver diseases have several characteristics of massive lipid accumulation and lipid metabolic disorder, and are divided into liver inflammation, liver fibrosis, liver cirrhosis (LC), and hepatocellular carcinoma (HCC) in patients. Interleukin (IL)-35, a new-discovered cytokine, can protect the liver from the environmental attack by increasing the ratio of Tregs (T regulatory cells) which can increase the anti-inflammatory cytokines and inhibit the proliferation of immune cellular. Interestingly, two opposite mechanisms (pro-inflammatory and anti-inflammatory) have connection with the ultimate formation of liver diseases, which suggest that IL-35 may play crucial function in the process of liver diseases through immunosuppressive regulation. Besides, some obvious advantages also imply that IL-35 can be considered as a new therapeutic target to control the progression of liver diseases, while its mechanism of function still needs further research.

## Highlights

• Effective and safe treatment is urgently needed, owing to the high mortality of the liver diseases.
• IL-35, a new-discovered cytokine, can protect liver from environmental attack by increasing the ratio of Tregs and inhibiting the immune response.
• IL-35 can be considered as a potential target in the treatment of liver diseases.

## Introduction

IL-35, a newly discovered heterodimeric cytokine and a member of the Interleukin (IL)-12 cytokine family (Table 1) (Li et al., 2019), consists of two subunits: α chain (p35) and β chain (EBI3) (Kong et al., 2016). In detail, p35 had dissociation between p35 and p40 gene regulation (Skrombolas et al., 2015). EBI3 was a homologue to p40 and the ciliary neurotrophic factor receptor, and its expression level was independent on EBV infection in B lymphoblastoid cells (Wetzel et al., 2020). Meanwhile, IL-35 was composed by heterodimer of IL-12Rβ2 chain and gp130 chain or homodimers of each chain, which subsequently activated the STAT signaling pathway (Terayama et al., 2014). Recent researches have confirmed that IL-35, mainly secreted by immune cells, including the newly reported Breg cells (CD4+CD25+), had a high expression level in the bone marrow, thymus, blood and liver tissues (Li et al., 2020b; Zhang et al., 2020). Functional analysis suggested that IL-35 played a crucial role in many autoimmune diseases and allograft rejection, such as rheumatoid arthritis (RA) (Li et al., 2019), multiple sclerosis (MS) (Badihian et al., 2018), systemic lupus erythematosus (SLE) (Ye et al., 2019), inflammatory bowel disease (IBD) (Su et al., 2018), psoriasis and experimental autoimmune uveitis (EAU) (Zhang et al., 2016). What’s more, it has been confirmed that IL-35 had a higher expression level in chronic hepatitis patients and its expression level had a positive association with the severity of infection (Dong et al., 2020). Additionally, IL-35 could reduce liver regeneration and hepatocyte proliferation both *in vivo* and *in vitro* (Yang et al., 2020b). Thus, it was possible that IL-35 might act as a potential target in treating liver diseases.

**TABLE 1:** The role of IL-12 family member.

Cytokine	Subunits	Receptor	Biological activity	Ref.(PMID)
IL-12	p35, p40	IL-12R-β1, IL-12R-β2	Induce differentiation of naïve CD4+ T cells into Th1 cells, facilitate Th1 differentiation, antagonize Th2 cell polarization and the production of IL-4 and IL-13, secret IFN-γ from T cells, NK cells, DCs and macrophages.	32132181, 32121641
IL-23	p19, p40	IL-23R, IL-12R-β1	Promote the maintenance of Th17 cells phenotype, stimulate the production of IL-17 from neutrophils and the production of IL-1β, TNF-α and IL-6 stabilize IL-17 expression	32185257, 32169850
IL-27	p28, EBi3	IL-27R, gp130	Induce differentiation of Th1 and Th2 cells, suppress the Th17 polarization induced by IL-10, IL-6 and TGF-β in combination with IFN-γ	32164467, 32120343
IL-35	Ebi3, p35	IL-12R-β2, gp130	Increase the generation of induced Treg population and nTreg activation, inhibit the proliferation of Th17 cells and Th1 cells, suppress expension of T cell	32169441, 32156533

As we all known, the incidence of liver disease was increasing year by year (Chen et al., 2020b). Frequently, liver diseases caused by inflammatory reaction and oxidative stress could lead to the accumulation of extracellular matrix (ECM), which then progressed to hepatitis, fibrosis, cirrhosis, and even hepatocellular carcinoma (HCC) (Chen et al., 2020c). Of note, liver diseases were related to imbalance between the innate immune and adaptive immune (Mendez-Sanchez et al., 2020). Various immune cells (Treg cells, Th cells, CD4/CD8+ T cells, Kupffer cells, liver mononuclear cells) and inflammatory cytokines (TNF-α, interleukin, interferon) were produced in response to risk factors, such as viruses, high-fat eating habits, alcohol and drug (Roderburg et al., 2020). Additionally, related evidence showed that the immunoregulation of IL-35 required multiple immune regulatory factors, through cooperating IL-10 and TGF-β, and then present effective and maximal anti-inflammatory outcome (Slawek et al., 2020).

**FIGURE 1:**
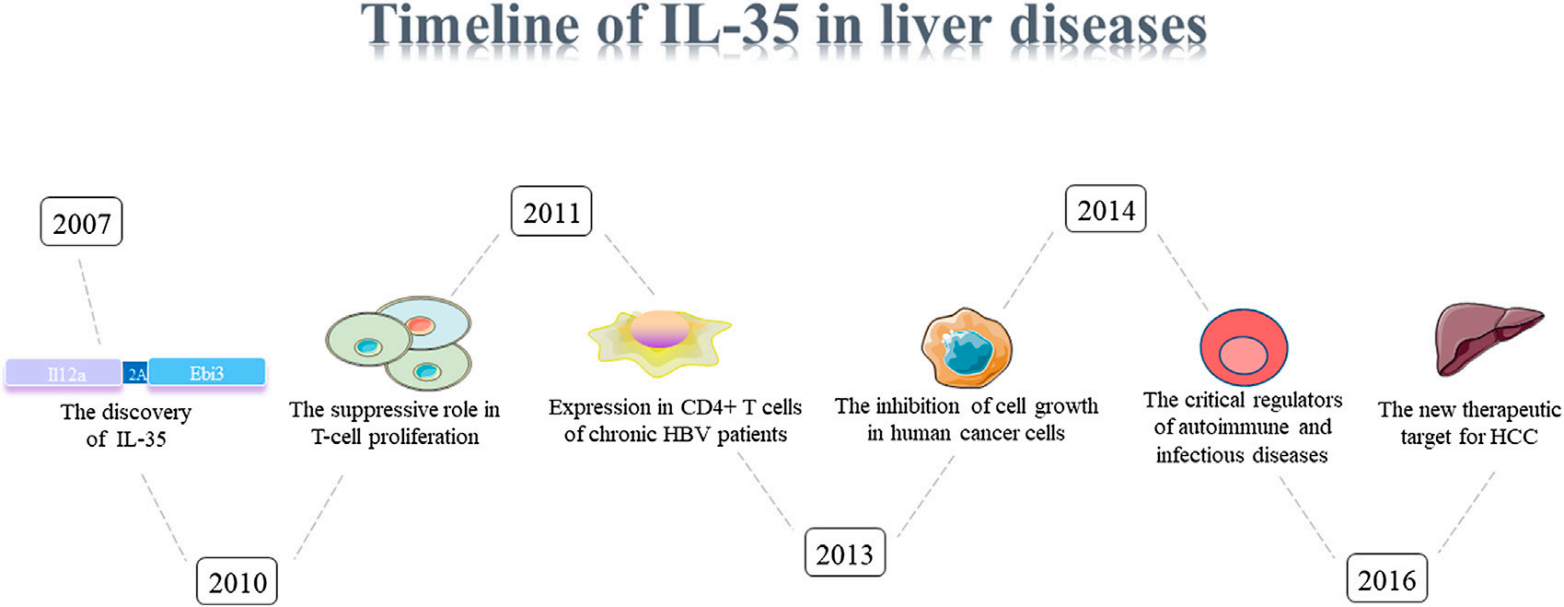
Timeline of IL-35.

**FIGURE 2:**
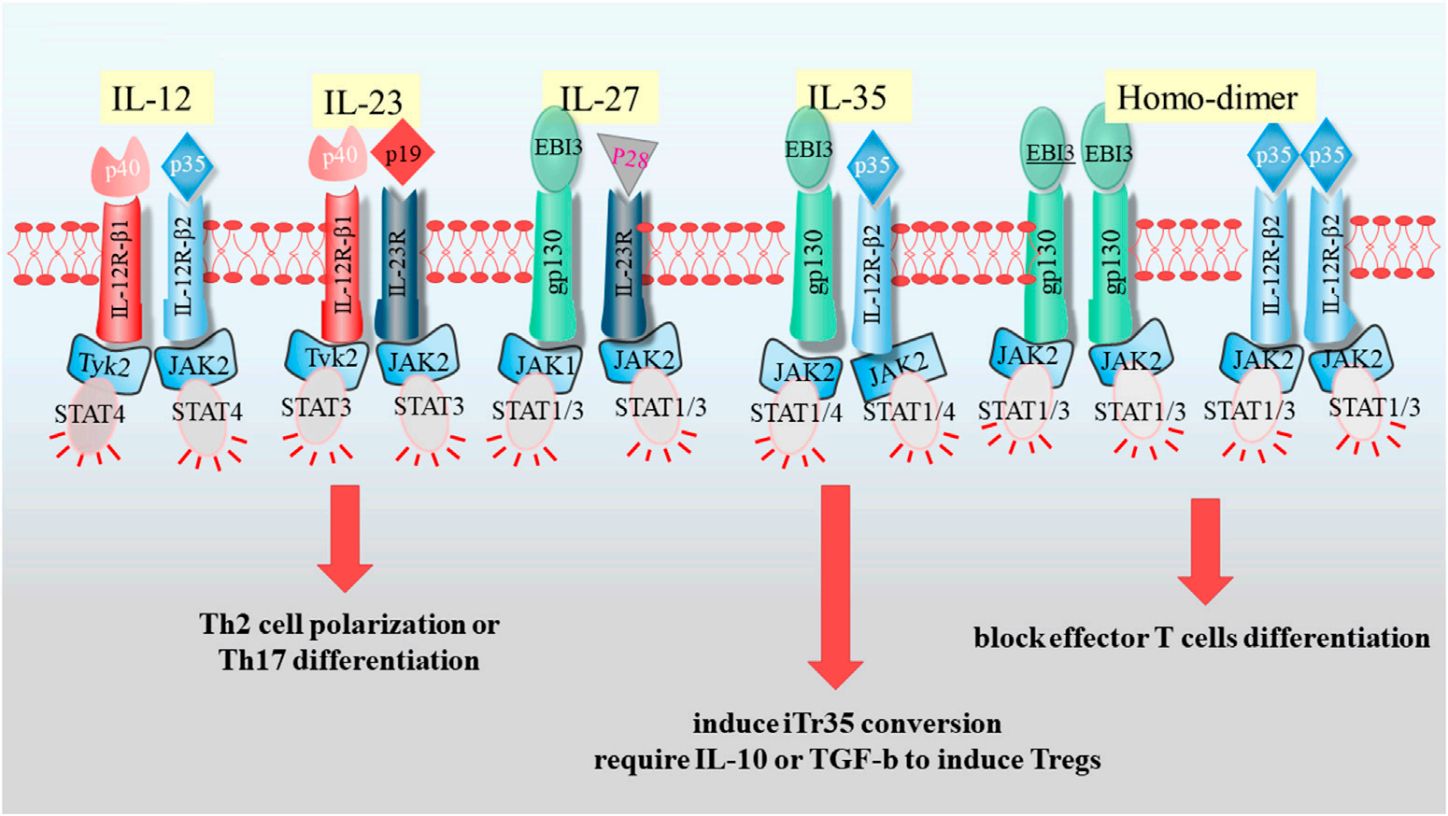
The relationship of IL-35 with liver diseases.

The pathogenesis of liver diseases was multifactorial, and the physiological function of IL-35 liver diseases in human were limited (Figures 1, 2). In this review, we systematically summarized the comprehensive roles of IL-35 on hepatitis, liver fibrosis, cirrhosis, HCC, as well as a therapeutic mediator for liver diseases.

## The Roles of Interleukin-35 in Liver Diseases

### Interleukin-35 and Acute Liver Injury Failure

ALF, also known as a life-threatening clinical syndrome of fulminant liver failure, has the common characteristic of the activation of inflammatory cells (Hoshino et al., 2020). The overall survival of ALF patients was 67%, and approximately 30% of these patients had an experience of liver transplantation operation (Mohamadi-Zarch et al., 2020). Of note, ALF was a global health problem due to poor clinical prognosis and high mortality. Apart from liver transplantation, few effective treatment existed (Dimitrova-Shumkovska et al., 2020). Interestingly, Yang et al. confirmed that, the serum IL-35 concentration of ALF was increased in ALF patients, along with exhausted peripheral CD8+ T cells (Yang et al., 2020b). Simultaneously, IL-35 stimulation was able to boost the expression levels of PD-1, CTLA-4, and LAG-3 in CD8+ T cells, but inhibit of perforin, granzyme B, and FasL expression levels (Yang et al., 2020b). Thus, the silence of IL-35 could treat ALF via targeting the PD-1, CTLA-4.

In a concanavalin A (Con A)-induced hepatitis mediating by T cell (Zhao et al., 2011), Bone marrow mesenchymal stem cells (MSCs) aggregate at the site of injury or inflammation, and show multi-directional differentiation potential (Wang et al., 2018b). IL-35 gene modified MSCs (IL-35-MSCs) was used to treat Con A-induced hepatitis and showed better therapeutic effect than the pure MSCs. IL-35-MSCs significantly decreased the expression level of FASL (Wang et al., 2018b). Furthermore, the INF-γ expression level was decreased and the protein expression of JAK1, STAT1 and STAT4 were increased significantly in the IL-35-MSCs transplanted groups (Hou et al., 2016). IL-35-MSCs regulated the inflammation through positively regulating JAK1/STAT1/4 signaling pathway and reducing INF-γ expression level. All in all, IL-35-MSCs played a protective role in Con A-induced hepatitis.

### Interleukin-35 and Hepatitis

Hepatitis was a common phenomenon in clinical work, including autoimmune hepatitis, alcoholic hepatitis, drug-induced hepatitis, bacterium hepatitis, viral hepatitis and others (Petta and Craxi, 2020). Generally, hepatitis B virus (HBV) and hepatitis C virus (HCV) were common in liver injury, both of which were non-cytopathic virus (Gonzalez and Gordon, 2020; Spyrou et al., 2020). It has been reported that IL-35 was associated with significant inflammation inhibition, which can inhibit the proliferation of T cell and the function f its effector (Li et al., 2020a). This compelling function pushed us to figure out the theoretical basis and other beneficial effects of IL-35 in the immune-pathogenesis of HBV, HCV hepatitis, and autoimmune hepatitis (AIH).

#### Interleukin-35 and Hepatitis B Virus

Hepatitis B virus, a common infectious disease worldwide, had a characteristic of high morbidity and mortality. According to the reports, about 240 million people worldwide were chronically infected with HBV (Cavallone et al., 2020; Li et al., 2020c). More than 700,000 people died from HBV-associated diseases each year (Bhuiyan et al., 2020). Asymptomatic carriers are more susceptible to liver cirrhosis and liver cancer. HBV mediated disease was related to different degrees of cell immune response, resulting in the development of severe hepatitis (Su et al., 2020). Furthermore, unsuccessful immune clearance of HBV will bring about chronic hepatitis and increase the risk of HCC (Li et al., 2020c). Thus, solving HBV infection was a crucial endpoint, and had a promising clinical outcome in HBV-induced disease.

Yang et al. confirmed that serum IL-35 was elevated in chronic hepatitis B virus (CHB). At the same time, studies found that Tregs and other cell types (including activated myeloid, endothelial cells, regulatory B cells) was capable to secret IL-35 (Yang et al., 2020a). Evidence showed that HBV could initiate CD4+ CD25+ Tregs to express IL-35 (Schneider et al., 2018; Yang et al., 2019a). The expression levels of EBI3, p35 and receptor of IL-35 were also highly expressed in CD4+ CD25+ Tregs of CHB patients. Notably, their expression levels were proved to be equal in activated and inactivated CD4+ CD25+ Tregs, which suggested that IL-35 could be secreted in activated and inactivated CD4+ CD25+ Tregs (Yang et al., 2019a). Additionally, the regulatory function of Tregs in EBI3−/− or p35 −/− mice was significantly inhibited compared to that of control group, implicating that IL-35 was vital for the activity of Treg’ functions. In terms of its function, IL-35 from Treg could block the differentiation of Th1 cells and Th17 cells away from excessive autoimmune reaction, thus preventing organisms like the liver from being immune to chronic infections triggered by virus damage (Teng et al., 2019; Yang et al., 2020a). Moreover, the proliferation of HBV-specific CTLs among CD8+ T cells were significantly lower in the peripheral blood mononuclear cells (PBMCs) after treated treatment with IL-35 (Dong et al., 2020). Interestingly, only high concentration of IL-35 (>40 ng/ml) could inhibit the expansion of CTLs and the secretion of IFN-γ, which was not related to the traditional CD4+ CD25+ Foxp3+ Tregs or other cytokines such as IL-10 and TGF-β (Dong et al., 2020). It was widely accepted that CD4+ T cell responsible for adaptive immune had many surface markers including CD45RA, CD45RO and CD25. HBV core antigen induced-CD45RA expression level was significantly decreased by IL-35 in total CD4+ T cells, showing that IL-35 has protective role in CD4+ T mediate adaptive immune in HBV attack. Additionally, the effect of IL-35 on innate immune response marked by CD11c (a DC marker) and CD86 (an activation marker of DC) were investigated (Yang et al., 2019a; Teng et al., 2019). IL-35 significantly decreased the proportion of CD11c+ cells in PBMCs, but the proportion of CD86 on CD11c+ cells increased slightly (Dong et al., 2020). Taken together, IL-35 might have significant impact on the disorder of immune-response regulation during HBV infection.

#### Interleukin-35 and Hepatitis C Virus

Investigation from the World Health Organization (WHO) announced that there were estimated 71 million people who had been infected by HCV globally (Elbedewy et al., 2020). The availability of safe and effective antiviral drugs to treat almost all HCV patients is now an important problem to be solved, posing a huge challenge to the public health (Kim et al., 2020). The survey indicated that only 7.4% of the diagnosed patients had received treatment, partly because of the economic barrier to taking highly effective direct-acting antiviral drugs (Martinello et al., 2020). Moreover, those patients recovering from treatment still remained the risk of reinfection. Despite effective direct-acting antiviral drugs (DAA) were introduced, there were still strong recommendations for preventive HCV vaccination (Darema et al., 2020; Martinello et al., 2020). Among the 73 HCV patients, the expression level of IL-35 in serum was observed to elevate in HCV patients. IL-35 was significantly decreased as a result of inhibiting viral replication. As expected, compared with healthy individuals, HCV patients had higher proportion of CD4+ CD25+ CD127dim/− Tregs in the population, meaning IL-35 had induced the differentiation of Tregs (Liu et al., 2017; Wei et al., 2018). Current studies have shown that IL-35 could cause persistent HCV infection, which may be an unfriendly aspect of IL-35, because increased Tregs inhibited antiviral immune activity. On the other hand, IL-35 also had a capability to protect HCV patients against liver injury by reducing the expression levels of pro-inflammatory cytokines (Liu et al., 2017). Therefore, the immunosuppressive role of IL-35 had a contradictory effect between the maintenance of viral persistence and the reduction of chronic inflammation in chronic HCV infection.

#### Interleukin-35 and Autoimmune Hepatitis

AIH was a necrotizing inflammatory liver disease associated with the interactive cell populations of the innate and adaptive immune response (Minaga et al., 2019). According to reports, the prevalence of AIH among women was gradually rising. If the patients could not be diagnosed or treated in time, AIH patients would rapidly progress to liver failure and even HCC (Liwinski et al., 2020). To be unfortunate, this remission therapy of conventional agents to AIH patients might cause serious side effects. There was an urgent need to design therapeutic agents with more specific and safer effect (Sandusadee et al., 2020). Of note, the expression level of IL-35 subunits (p35/EBI3) were upregulated in the liver of AIH patients. Lian et al. found that IL-35 subunits (EBI3 and p35) in AIH liver tissue had an increased expression level and a positive association with hepatitis and fibrosis. Moreover, the expression level of EBI3 was positively related to age, IgG and AST expression levels in serum, and negatively correlated with hemoglobin and albumin (Lian et al., 2019). In addition, it was metastatic plasmacytoid DCs (pDCs) from peripheral blood fight against virus in the liver, rather than resident pDCs. There was a higher expression level of IL-35 in the liver, suggesting that these IL-35-producing cells participated in the pathogenesis of AIH (Lian et al., 2019; Zhang et al., 2019). Meanwhile, it was also confirmed that the protective effect of pDCs was related to recruitment of Tregs in an IL-35-dependent way (Chen et al., 2020a). Sum up, it’s necessary to study the effect of IL-35 on immune function and provide theoretical basis for the treatment of AIH.

### Interleukin-35 and Liver Fibrosis

Liver fibrosis with a disrupted liver architecture was always attributed to the progressive accumulation and reduction of ECM remodeling (Khalil et al., 2020). The formation of liver fibrogenesis was regarded as a wound-healing response, which was driven primarily by various immune response to hurt parenchymal cells. Histological features of fibrosis caused by autoimmune diseases, included progressive intrahepatic bile duct destruction, cholestasis, and liver cirrhosis (LC) (Gao et al., 2020). Notably, liver fibrosis was found to be an intermediate stage between CHB and LC, which was also stressed to be a reversible process.

Up to date, the correlation of IL-35 and liver fibrosis was quite blurred and indistinct due to the limited relevant studies. It was hard to figure out the specific role of IL-35 in process of liver fibrosis. At present, the pathogenic role of Th17 cells in promoting liver injury and fibrosis was widely recognized (Peng et al., 2019). Interestingly, IL-35 suppressed the proliferation of CD4+ CD25− effector T cells and the differentiation of Th17 cells (Huang et al., 2019; Luo et al., 2019), supporting that the relief of severity of inflammation in liver injury due to the reduction of Th17 cells induced by IL-35. The knockout of IL-12p35 gene promoted Th17 cell response and inhibited Th1 cell response, suggesting that IL-35 might be closely related to liver fibrosis (Huang et al., 2019). Taking all the studies into account, IL-35 secreted from Tregs could suppress the activity of Th17 cells and decreased its amount.

### Interleukin-35 and Liver Cirrhosis

LC, a frequent and dangerous disease, caused numerous clinical contacts due to its complications (Fenster et al., 2020). This process brought about a progressive loss of liver function, such as catabolism of toxins and drugs, metabolism of carbohydrates, lipids and proteins. Moreover, the histological characteristics of LC included progressive liver dysfunction and replacement of liver tissue by fibrotic scar tissue and regenerating nodules (Ferrarese et al., 2020). The pathophysiology and treatment of LC were of high international standard and formed a qualified basis for rational clinical handling of LC patients. Primary biliary cirrhosis (PBC) was an autoimmune liver disease and LC, and mainly caused by the body’s autoimmune disorders (Xu et al., 2020b).

The expression level of IL-35 in serum in PBC patients was lower than that in healthy individuals. The expression level of Ebi3 mRNA was decreased in PBC patients as well, but no statistical difference in the expression level of p35 mRNA level (Li et al., 2018). IL-35 in PBC patients was inversely associated with expression levels of clinical parameters, such as ALP, GGT, AST, ALT, and cytokines produced by CD4+ T cells (IFN-γ, IL-23, IL-17). Meanwhile, at different stages of disease, IL-35 was positively correlated with the TGF-β closely, which was related to Treg expression level in LC patients. Interestingly, the change trend of the expression levels of TGF-β and IL-35 in serum was consistent with Th17 Tregs in PBC (Li et al., 2018). Studies shown that the expression level of IL-35 might have relevance to histological grade and the severity of PBC. The expression level of IL-35 in serum in patients with stage III or IV was higher than that in patients with stage II. Furthermore, the mRNA and protein expression levels of IL-35 had negative association with LC Child-Pugh classification (Ma et al., 2014; Li et al., 2018). LC patients with portal hypertension were treated with phased joint operation (Cho et al., 2020). Then, the concentrations of IL-35, IL-6 and IL-17 in peripheral blood were significantly reduced after intervention. Whereas, EBI3 mRNA expression levels in postoperative group were higher than normal control group (Wang et al., 2014).

In the early phase of HBV-related LC patients, Sun et al. have observed an increase in population of Th17 cell and activation of hepatic stellate cell secreting pro-inflammatory cytokines (IL-17, TGF-β and IL-22) in LC patients. In order to reduce the damage of hepatocytes by cytokines, IL-35 and IL-10 subsequently secreted from Treg significantly blocked the differentiation of Th17 cells and the production of IL-17 in the blood specimen of HBV-related LC patients (Shi et al., 2015). In addition, IL-35 knockout promoted the expression levels of IL-17 and IL-22 (Ming et al., 2015). IL-22 is a very interesting cytokine, which has also play very important in liver diseases (Hwang et al., 2020). Mechanically, IL-35 could prevent the binding of TGF-β and its receptor, which inhibit phosphorylation of Smad3 a downstream effector of TGF-β receptor, thereby preventing the differentiation of Th17 cells and the synthesis of IL-17 in the blood of HBV-related LC patients (Ming et al., 2015). Taken together, IL-35 is an immunosuppressive molecule that may participate in the pathological process of PBC by inhibiting the expression levels of Th1/Th17-related cytokines and inducing the expression levels of Treg-related cytokines.

### Interleukin-35 and Hepatocellular Carcinoma

HCC, namely liver cancer, could be divided into primary hepatoma and secondary hepatoma. Primary hepatoma originated in the liver epithelial or mesenchymal tissue, and had the characteristics of high incidence and high malignancy (Hwang et al., 2020). Secondary or metastatic hepatoma would happen once liver tumor was from other organs (such as stomach, pancreas, colorectal, ovarian, uterine). HCC in China accounted for the first and second cause of death in rural and urban areas, respectively.

Increasing evidence showed that IL-35 could play a significant role in the development and progression of HCC. The expression level of IL-35 in serum was increased in patients with non-viral hepatitis-related HCC (Tsai et al., 2020). The results of tissue microarray in 75 HCC patients confirmed that IL-35 was mainly distributed in the cytoplasm of cancer cells and peritumoral hepatocytes. In addition, higher histological grades, larger tumor size, positive microvascular invasion and lymph node/distant metastasis were associated with low expression level of IL-35 in HCC patients. It is suggested that down-regulated the expression level of IL-35 could improve the poor prognosis of HCC (Tsai et al., 2020).

It was reported that IL-35 derived from Tregs could facilitate tumor growth by leading to T cell exhaustion, thereby showing a limitation in anti-tumor immunity. Yang et al. found that IL-35 declined peripheral perforin mRNA and granzyme mRNA of CD8+ T cells in peripheral and liver-resident from HCC patients. Whereas, the exhausted markers of PD-1 and CTLA-4 were highly expressed in CD8+ T cells treated with IL-35, meaning that multiple cytotoxicity mechanism of CD8+ T cells were applied to therapy HCC (Li et al., 2016). HepG2 cells with overexpressed IL-35 had higher HLA-ABC and CD95, and lower activities of MMP-2 and MMP-9 responsible for degradation of the basement membrane and the ECM, finally presenting decreased cell migration, invasion and single cell colony formation abilities (Yang et al., 2019b). Additionally, the Notch signaling pathway was activated in HCC cells and positively correlated with cell invasive migration capabilities, suggesting that Notch signaling pathway was possibly involved in the migration of invasive HCC cells (Tsai et al., 2020). Moreover, the combination of IL-17 and IL-35 could inhibit migration of different invasive hepatoma cells, including HepG2, SMMC-7721 and MHCC97H cells. Meanwhile, IL-35 could increase the expression level of E-cadherin and decrease that of Snail, which was the major reason of cell adhesion and tumor escape (Long et al., 2016). Above all, these results supported that the decreased IL-35 in HCC tissues might result in the progression of HCC through multiple anti-tumor immune mechanisms.

## The Regulatory Role of Interleukin-35 in Liver Diseases

It was well acknowledged that IL-35 had an inhibitory impact on immune system by stimulating and expanding Tregs (Qiu et al., 2016). Evidences showed that IL-35 expression was obviously elevated in HCV patients, and had a positive association with DNA and RNA load of virus (Liu et al., 2017). Langhans et al. have found that the viral core antigen could induce the production of specific Tregs in chronic viral infection patients (Xing and Tian, 2020). Treg cells comprised 5% to 10% of CD4+ T cells. It had two main classification: natural CD4+ CD25+ Treg cells (nTreg) originated from thymus and CD4+ CD25-T cells (iTreg) derived from native CD4+ T cell precursors after IL-10, TGF-β and IL-35 stimulation (Tumino et al., 2017). Gene knockout or IL-35 inhibitor was able to rescue nTreg mediated by immune suppression. Furthermore, it was observed that IL-35 also suppressed proliferation of T cell through a cell cycle arrest at G1 phase, without affecting apoptosis of T cell, indicating the important reason of persistent chronic infection (Bardel et al., 2008). Additionally, blockage of viral replication resulted in decreased level of IL-35 in serum (Table 2). Therefore, the change of the ratio in Treg/T cells or anti-/pro-inflammatory cytokines might be seen as indicators or prognostic markers to evaluate the therapeutic effectiveness.

**TABLE 2:** Functional role of IL-35 in different liver diseases.

Types of diseases	Expression of IL-35	Functions	Ref. (PMID)
HBV	+	Prevente the hepatocytes apoptosis by reducing the FASL expression, decrease secretion if IFN-γ by the JAK1-STAT1/STAT4 signal pathway	28644966
HCV	+	Increase the proportion of Foxp3+ Tregs in patients with chronic hepatitis, reduce IFN-γ, IL-6, IL-8, and TNF-α by hepatocytes by p-STAT1 and p-STAT3 in HCV-infected hepatocyte	20222873, 30067145
Fibrosis	+	Deletion of IL-12p35 subunit induce a Th17 cell response, inhibited a Th1 cell response, and caused liver fibrosis	22576253
Liver cirrhosis	−	Serum IL-35 levels were negatively correlated with the LC Child-Pigh classification plasma IL-35 from PBC patients was negatively correlated with levels of IFN-γ, IL-23, and IL-17, and positively correlated with TGF-β in the early and late stages	25323532, 29445068
HCC	+	Inhibited perforin mRNA expression and IFN-γ production in CD8+ T cells from non-viral hepatitis-related HCC, suppressed cytolytic and non-cytolytic activity of CD8+ T cells	31134088, 27002937

As known, Kupffer cells (KC) was responsible for inflammation and elimination of pathogens. Inflammatory Mφ, commonly called M1 and phenotypically described as CD40+ CD86+ HLA− DR+ was characterized by secreting pro-inflammatory cytokines or chemokines, like IL-1β and nitric oxide (NO) (Long et al., 2013; Boltjes et al., 2015). Anti-inflammatory Mφ, also termed M2, was consider to express the arginase 1, mannose receptors, and the high affinity scavenger receptor CD163, and release anti-inflammatory cytokines, such as like IL-10, IL-35 and TGF-β, as well as angiogenic agents, like VEGF (Coelho and Drummond, 2019). M2 were responsible for the clearness of inflammation in tolerance mechanisms (Shigeoka et al., 2020). When KCs were exposed to LPS, IL-35 could alleviate the lethality of KCs toward hepatocytes by reducing pro-inflammatory cytokines, like NO, TNF-α, IL-1β and IL-6, along with boosting anti-inflammatory cytokines (IL-4 and IL-10) (Shao et al., 2018). Meanwhile, it was showed that IL-35 had no impact on production of NO and TNF-α in response to LPS treatment in deletion of IL-10 mice and KCs *in vitro*.

Besides, IL-35 was recognized as an indispensable molecule in suppressing Th1/Th17 cells and weakening immune response to pathogen. IL-35 could partly attenuate chronic liver inflammation by inhibiting IL-17 production and Th17 differentiation from CD4+ T cells *in vitro*, and reducing the amount of Th17 cells of liver *in vivo* (Zheng et al., 2018; Yang et al., 2019a). Ye et al. found that IL-17-prodocing cells increased in liver fibrosis (Teng et al., 2019). Additionally, the increase in the number of Th17 cells in liver cirrhosis patients could facilitate activation of HSC, which contribute to further progression of liver (Ye et al., 2010). The combination of IL-17 monoclonal antibody with IL-35 could suppress the invasie Notch signalling pathway.ve migration capability of hepatoma cells.

Notably, it was reported that MSCs modified by IL-35 (IL-35- MSCs) could specifically move to the injured local tissues in liver, significantly reduce the necrotic areas in livers, and block the apoptosis of hepatocytes by reducing the FASL and mononuclear cell (MNC) (Wang et al., 2018b). Another study showed that IL-35-MSCs could decrease the secretion of IFN-γ from liver MNC, which was related to the JAK1-STAT1/STAT4 signalling pathway (Shouse and Nikolaenko, 2019). To sum up, the construction of humanized antibodies that IL-35 might provide benefits for liver diseases therapy.

## The Therapeutic Potential Role of Interleukin-35

**FIGURE 3:**
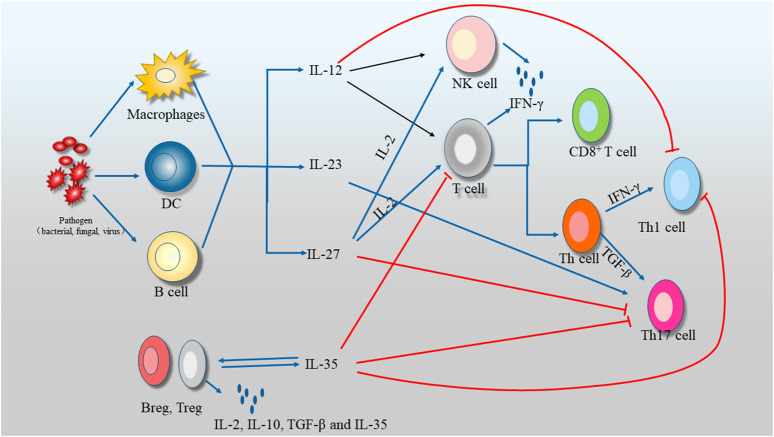
The regulated role of IL-12 family in Immunity (Tregs: T regulatory cells).

IL-35 was considered to serve as specific diagnostic markers, potential therapeutic targets and strong indicators of prognosis, due to the large heterogeneity of its clinical, laboratory and histological features. High expression level of IL-35 was found in various tumor tissues, including pancreatic ductal adenocarcinoma (Lan et al., 2019), colorectal cancer (Huang et al., 2017), acute myeloid leukaemia (Wang et al., 2018a) and non-small-cell lung cancer (Wang et al., 2015). Elevated expression level of IL-35 in serum in HCC patients might act as an independent prognostic indicator (Yang et al., 2019b). Furthermore, circulating IL-35 expression level might suggest the dysfunctional immune system in anti-tumor response, showing the activation of Tregs and the apoptosis of CD8+ T cell (Heim et al., 2019). Moreover, IL-35 expression level was positively correlated with intrahepatic cholangiocarcinoma (ICC) aggressiveness, and served as an independent prognostic factor (Xing and Tian, 2020). IL-35R was composed by heterodimer of IL-12Rβ2 chain and gp130 chain or homodimers of each chain. Luckily, it was noted that gp130 had a better accuracy to predict the survival and recurrence of ICC patient compared with the traditional TNM stage (Xing and Tian, 2020). The expression of IL-35 or gp130 was an independent prognostic factor in IL-35-mediated ICC (Zhang et al., 2018; Xing and Tian, 2020). Moreover, IL-35 was reported to promote the differentiation of pro-inflammatory liver MΦ, and accelerate the shift from M1 to M2, suggesting that IL-35 was a beneficial target to break immune tolerance and facilitate a functional cure of chronic viral infections (Figure 3).

In addition, although the induction of new receptor-based drugs has been put forward and has attracted massive discussion in recent years, obstacles still remain such as tumor cells still sharing some antigens with normal cells. For example, tumor cells still share some antigens with normal cells. It is not evitable that anti-infection curation can trigger severe autoimmune reactions. Thus, it has long way to find out a unique, safe and efficacious approach to treat liver diseases.

## Conclusion

IL-35 played crucial function in the process of liver diseases through immunosuppressive regulation. Pro-inflammatory and anti-inflammatory, two opposite mechanism, were in relation to the ultimate formation of diseases at the occurrence of liver injury (Figure 2).

The problem between immune-mediated pathogen clearness and inflammation in our organism was a meaningful thing to explore in liver injury (Zhou et al., 2020). Early inflammation was a protective mechanism for liver to fight against exogenous materials. Unfortunately, inflammation could also be caused by both strong or weak clearance. Specifically, the decrease of immune activity would increase the chance of cancer and chronic infection, and the main cause of pathological evolution of various acute infectious diseases, allergic reactions and autoimmune diseases was the over-active immune system (Roth et al., 2020; Yoshimura et al., 2020). Considering the interaction between various innate immune and adaptive immune, the environment of cytokines and chemokines secreted by immune cells was extremely important for the fate of these cells, which played a role of isolation or mutual adjustment (Yoshimura et al., 2020). Practical experiments had proved the expression level of IL-35 in serum in patients with PBC were low (Li et al., 2018). In addition, lower IL-35 was also observed in CHB patients, accompanied by more Tregs and more CD4+ T cells in high viral load group than healthy controls (Liwinski et al., 2020). However, the expression level of IL-35 in serum was higher in HepG2 cells, to which was conducive to evading attack from the immune system (Zheng et al., 2018). Furthermore, IL-35 required additional immune-regulatory cytokines, such as IL-10, TGF-β, IFN-γ to induce effective and maximal anti-inflammatory and immunomodulatory effects (Xu et al., 2020a). Thereby, the important balance among regulatory T cells, T helper cells and T cells was determined by IL-35 in cooperating with other cytokines, which affected inflammation, pathogen clearness and tumor escape.

## Future Expectation

As we all known, liver disease is one of the most prevalent diseases all around the word, with a high incidence rate and mortality rate (Zhang et al., 2020). On the basis of the statistics of the WHO, this puts people at high risk of death due to LC and HCC, resulting in a heavy global health burden (Bayoumi et al., 2020). If the local injury is persistent and intense, normal liver tissue will be gradually replaced by nonfunctional fibrotic scars due to the maintained inflammatory response. The imbalance between tissue regeneration and fibrosis will determine the outcome of health recovery or cirrhosis.

**FIGURE 4:**
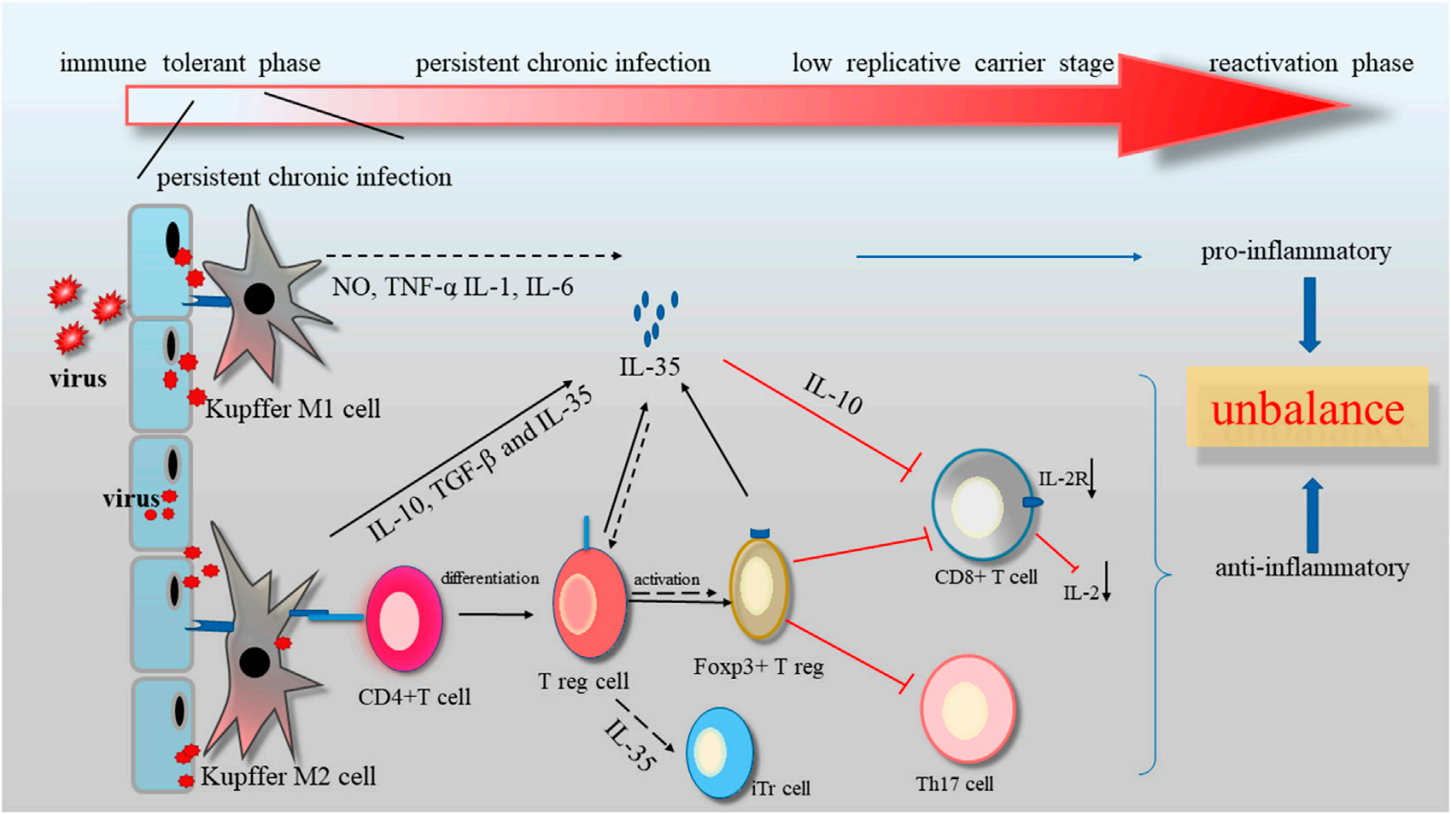
The related structures and signals of IL-12 family (IL-12, IL-23, IL-27 and IL-35).

In normal physiological, the main physiological function of IL-35, mainly secreted by Treg, is to inhibit the formation of Th1 and Th17 cells and promote the proliferation of Treg (Lim et al., 2020). Treg, a special subset of CD4+ T cells, is essential in the regulation of immune-mediated diseases such as autoimmune diseases, tumorigenesis and some infections (Bobryshev et al., 2015). Foxp3+ Treg is the activated form of Treg. Foxp3+ Treg becomes an important infection tolerance regulator by transforming conventional CD4+ foxp3− T cells (Tconv cells) into induced CD4+ Foxp3+ (Dehghani et al., 2020). Treg is directly produced by suppressive cytokines, such as IL-10, TGF-β, and IL-35, or indirectly produced through activating dendritic cells. IL-35-induced Tregs derives from CD4+ Foxp3− T cells treated with IL-35, termed “iTr35 cells,” which has strongly suppressive ability *in vivo*, and mediated immunosuppressive function via IL-35 but not via the inhibitory TGF-β or IL-10 (Zhang et al., 2019). Unconventionally, the transcription factor Foxp3 is not expressed or required by the newly identified iTr35 cells. Moreover, IL-35 and iTr35 cell develope a positive feedback loop to interact each other (Hamed et al., 2019; Xu et al., 2020b). It is found that HBV could also serve as a stimulus to the Treg to initiate IL-35. IL-35 is expressed in different cell types, diseases, or developmental stages to execute a wide variety of regulatory roles at virtually gene expression and translation (Bianco et al., 2020; Cavallone et al., 2020). However, most normal T cell subsets and tissues seem not to constitutively express IL-35 in human. Furthermore, the regulatory activity of Treg from EBI3 or p35 knockout mice was significantly reduced, compared to that of wild-type Treg *in vivo* and *in vitro*, suggesting that IL-35 was critical for the regulatory activity of Treg (Huang et al., 2019; Tsai et al., 2020) (Figure 4).

Monoclonal antibodies (mAbs) excellent drug candidates due to their high antigen specificity, also have favorable PK properties and engineer-friendly modular structure compared to small molecules (Sullivan et al., 2020). Beginning with the approval of the first monoclonal antibody rituximab for hematological tumor therapy in 1997, this targeted therapy branch has been shown to significantly enhance tumor therapy, bringing high specificity and minimal off-site toxicity (Kovalova et al., 2020). Meanwhile, previous investigations have confirmed that IL-35 plays an important role in liver diseases. Preparation of IL-35 monoclonal antibody in the treatment of liver diseases may promote liver healing and reduce side effects. On the other hand, CRISPR-Cas9 genome editing system provides a tremendous promising strategy for versatile and high specifically targeting genome editing (Popkova et al., 2020). Genome editing also involves the precise manipulation of cellular DNA sequences to alter cell fates and organism traits, that has been widely applied in treating various genetic diseases (Deng et al., 2020). Generally, genome editing strategies involve DNA modifications in living organisms, including “beneficial” deletions, corrections via gene replacement, and insertions, and related protocols have been established for introducing these modifications in stem cells during the last decades (Doudna, 2020). Therefore, genome editing strategy is likely to treat liver diseases by changing the expression level of IL-35.

Due to the lack of treatment options in liver diseases, there is an urgent need to improve our understanding of the mechanisms of liver diseases so that more effective treatments can be developed to prevent and combat liver diseases. Overall, current evidence supported that IL-35 is a hallmark of immune-regulation in chronic hepatitis, liver fibrosis, LC and HCC progression.

## Author Contributions

SH: Design of the work, drafting the manuscript; P-pL and YH: Analysis and critical revision of the article, design of figures and tables; X-yZ, S-wJ, QM, L-yL, and J-fY: Review of traditional oriental medicine herbs in different sources, reviewing the pharmacological mechanisms; LY, H-yG; C-cY; X-mM and JL: Reviewing the auditory relevance; H-wL, TX, and HZ: Analysis of ethnopharmacological relevance, and final approval. All authors listed have made a substantial, direct, and intellectual contribution to the work and approved it for publication.

## Funding

This project was supported by the National Natural Science Foundation of China (Nos. 81700522, 81602344), Natural Science Foundation of Anhui Province (1808085MH235), 512 Talent Cultivation Plan of Bengbu Medical College (by51201315), Natural Science Foundation of Anhui Province (2008085QH401).

## Conflict of Interest

The authors declare that the research was conducted in the absence of any commercial or financial relationships that could be construed as a potential conflict of interest.

## Abbreviations

LC, liver cirrhosis; HCC, hepatocellular carcinoma; IL-35, Interleukin-35; EBI3, Epstein–Barr virus-induced gene 3; gp130, glycoprotein130; STAT, signal transducer and activator of transcription; Tregs, T regulatory cells; ECM, extracellular matrix; HSCs, hepatic stellate cells; KCs, Kupffer cells; TNF-α, tumor necrosis factor-α; TGF-β, transforming growth factor-β; PD-1, programmed death-1; CTLA-4, cytotoxic T lymphocyte antigen 4; IL-35-MSCs, IL-35-modified MSCs; JAK1, Janus kinase 1; HBV, hepatitis-B virus; HCV, hepatitis C virus; AIH, autoimmune hepatitis; PBMCs, peripheral blood mononuclear cells; PBC, Primary biliary cirrhosis; MMP-2, matrix metalloproteinase-2; mAbs, Monoclonal antibodies.

## References

[B1] Badihian S. Shaygannejad V. Soleimani P. Mirmosayyeb O. Samee Z. Manouchehri N. (2018). Decreased serum levels of interleukin-35 among multiple sclerosis patients may be related to disease progression. J. Biol. Regul. Homeost. Agents. 32 (5), 1249–1253. 30334421

[B2] Bardel E. Larousserie F. Charlot-Rabiega P. Coulomb-L’Hermine A. Devergne O. (2008). Human CD4+ CD25+ Foxp3+ regulatory T cells do not constitutively express IL-35. J. Immunol. 181 (10), 6898–6905. 10.4049/jimmunol.181.10.6898 18981109

[B3] Bayoumi A. Gronbaek H. George J. Eslam M. (2020). The epigenetic drug discovery landscape for metabolic-associated fatty liver disease. Trends Genet. 36 (6), 429–441. 10.1016/j.tig.2020.03.003 32396836

[B4] Bhuiyan A. R. Kabir N. Mitra A. K. Ogungbe O. Payton M. (2020). Disparities in hepatitis B vaccine coverage by race/ethnicity: the national health and nutrition examination survey (NHANES) 2015-2016. Diseases. 8 (2), 10 10.3390/diseases8020010 PMC7348843 32316174

[B5] Bianco C. Jamialahmadi O. Pelusi S. Baselli G. Dongiovanni P. Zanoni I. (2020). Non-invasive stratification of hepatocellular carcinoma risk in non-alcoholic fatty liver using polygenic risk scores. J. Hepatol. S0168–8278 (20), 33811–33813. 10.1016/j.jhep.2020.11.024 PMC7987554 33248170

[B6] Bobryshev Y. V. Sobenin I. A. Orekhov A. N. Chistiakov D. A. (2015). Novel anti-inflammatory interleukin-35 as an emerging target for antiatherosclerotic therapy. Curr. Pharmaceut. Des. 21 (9), 1147–1151. 10.2174/1381612820666141014123810. 25312725

[B7] Boltjes A. van Montfoort N. Biesta P. J. Op den Brouw M. L. Kwekkeboom J. van der Laan L. J. (2015). Kupffer cells interact with hepatitis B surface antigen *in vivo* and *in vitro*, leading to proinflammatory cytokine production and natural killer cell function. J. Infect. Dis. 211 (8), 1268–1278. 10.1093/infdis/jiu599 25362194

[B8] Cavallone D. Ricco G. Oliveri F. Colombatto P. Moriconi F. Coco B. (2020). Do the circulating Pre-S/S quasispecies influence hepatitis B virus surface antigen levels in the HBeAg negative phase of HBV infection? Aliment. Pharmacol. Ther. 51 (12), 1406–1416. 10.1111/apt.15753 32390175

[B9] Chen C. Chencheng Z. Cuiying L. Xiaokun G. (2020a). Plasmacytoid dendritic cells protect against middle cerebral artery occlusion induced brain injury by priming regulatory T cells. Front. Cell. Neurosci. 14, 8 10.3389/fncel.2020.00008 32076400 PMC7006436

[B10] Chen H. T. Huang H. L. Li Y. Q. Xu H. M. Zhou Y. J. (2020b). Therapeutic advances in non-alcoholic fatty liver disease: a microbiota-centered view. World J. Gastroenterol. 26 (16), 1901–1911. 10.3748/wjg.v26.i16.1901 32390701 PMC7201149

[B11] Chen W. Yang A. Jia J. Popov Y. V. Schuppan D. You H. (2020c). Lysyl oxidase (LOX) family members: rationale and their potential as therapeutic targets for liver fibrosis. Hepatology. 72 (2), 729–741. 10.1002/hep.31236 32176358

[B12] Cho H. C. Kim J. H. Cha R. R. Kim W. S. Lee J. M. Lee S. S. (2020). Clinical significance of endothelial progenitor cells in patients with liver cirrhosis with or without hepatocellular carcinoma. Eur. J. Gastroenterol. Hepatol. 32 (1), 87–94. 10.1097/MEG.0000000000001484 31790004

[B13] Coelho C. Drummond R. A. (2019). Kupffer cells mediate systemic antifungal immunity. Trends Immunol. 40 (12), 1071–1073. 10.1016/j.it.2019.11.001 31735512 PMC7960509

[B14] Darema M. Cholongitas E. Filiopoulos V. Marinaki S. Pavlopoulou I. D. Tsoubou I. (2020). Efficacy and safety of new direct-acting antivirals in kidney transplant recipients with chronic hepatitis C: a single-center study. Ann. Gastroenterol. 33 (3), 285–292. 10.20524/aog.2020.0481 32382232 PMC7196623

[B15] Dehghani M. Kalani M. Golmoghaddam H. Ramzi M. Arandi N. (2020). Aberrant peripheral blood CD4(+) CD25(+) FOXP3(+) regulatory T cells/T helper-17 number is associated with the outcome of patients with lymphoma. Cancer Immunol. Immunother. 69 (9), 1917–1928. 10.1007/s00262-020-02591-y 32385519 PMC11027683

[B16] Deng H. Tan S. Gao X. Zou C. Xu C. Tu K. (2020). Cdk5 knocking out mediated by CRISPR-Cas9 genome editing for PD-L1 attenuation and enhanced antitumor immunity. Acta Pharm. Sin. B. 10 (2), 358–373. 10.1016/j.apsb.2019.07.004 32082979 PMC7016277

[B17] Dimitrova-Shumkovska J. Krstanoski L. Veenman L. (2020). Potential beneficial actions of fucoidan in brain and liver injury, disease, and intoxication-potential implication of sirtuins. Mar. Drugs. 18 (5). 10.3390/md18050242 PMC7281157 32380741

[B18] Dong Y. Li X. Yu Y. Lv F. Chen Y. (2020). JAK/STAT signaling is involved in IL-35-induced inhibition of hepatitis B virus antigen-specific cytotoxic T cell exhaustion in chronic hepatitis B. Life Sci. 252, 117663 10.1016/j.lfs.2020.117663 32302624

[B19] Doudna J. A. (2020). The promise and challenge of therapeutic genome editing. Nature. 578 (7794), 229–236. 10.1038/s41586-020-1978-5 32051598 PMC8992613

[B20] Elbedewy T. A. Elashtokhy H. E. A. Abd-Elsalam S. Suliman M. A. (2020). Hepatitis C virus infection and treatment as independent prognostic factors in diffuse large B-cell lymphoma Egyptian patients. Curr. Cancer Drug Targets. 20 (8), 638–645. 10.2174/1568009620666200511084731 32392114

[B21] Fenster M. Alayo Q. A. Khatiwada A. Wang W. Dimopoulos C. Gutierrez A. (2020). Real-world effectiveness and safety of tofacitinib in Crohn’s disease and IBD-U: a multicenter study from the TROPIC consortium. Clin. Gastroenterol. Hepatol. S1542–3565 (20), 31438–31445. 10.1016/j.cgh.2020.10.025 PMC8044250 33068786

[B22] Ferrarese A. Burra P. Senzolo M. (2020). A commentary on the interplay between severity of liver disease and bacterial infection in hospitalized patients with cirrhosis. Dig. Dis. Sci. [Epub ahead of print]. 10.1007/s10620-020-06278-3 32385702

[B23] Gao J. Wei B. de Assuncao T. M. Liu Z. Hu X. Ibrahim S. (2020). Hepatic stellate cell autophagy inhibits extracellular vesicle release to attenuate liver fibrosis. J. Hepatol. 73 (5), 1144–1154. 10.1016/j.jhep.2020.04.044 32389810 PMC7572579

[B24] Gonzalez H. C. Gordon S. C. (2020). Hepatitis C: does successful treatment alter the natural history and quality of life? Gastroenterol. Clin. N. Am. 49 (2), 301–314. 10.1016/j.gtc.2020.01.007 32389364

[B25] Hamed F. N. Astrand A. Bertolini M. Rossi A. Maleki-Dizaji A. Messenger A. G. (2019). Alopecia areata patients show deficiency of FOXP3+CD39+ T regulatory cells and clonotypic restriction of Treg TCRbeta-chain, which highlights the immunopathological aspect of the disease. PLoS One. 14 (7), e0210308 10.1371/journal.pone.0210308 31277078 PMC6611701

[B26] Heim L. Kachler K. Siegmund R. Trufa D. I. Mittler S. Geppert C. I. (2019). Increased expression of the immunosuppressive interleukin-35 in patients with non-small cell lung cancer. Br. J. Canc. 120 (9), 903–912. 10.1038/s41416-019-0444-3 PMC6734661 30956278

[B27] Hoshino K. Nakamura Y. Nakano T. Watanabe A. Sheng H. Tachibana K. (2020). Enhanced effect of recombinant human soluble thrombomodulin by ultrasound irradiation in acute liver failure. Sci. Rep. 10 (1), 1742 10.1038/s41598-020-58624-0 32015385 PMC6997189

[B28] Hou C. Wu Q. Ouyang C. Huang T. (2016). Effects of an intravitreal injection of interleukin-35-expressing plasmid on pro-inflammatory and anti-inflammatory cytokines. Int. J. Mol. Med. 38 (3), 713–720. 10.3892/ijmm.2016.2688 27460435 PMC4990317

[B29] Huang C. Li N. Li Z. Chang A. Chen Y. Zhao T. (2017). Tumour-derived Interleukin 35 promotes pancreatic ductal adenocarcinoma cell extravasation and metastasis by inducing ICAM1 expression. Nat. Commun. 8, 14035 10.1038/ncomms14035 28102193 PMC5253665

[B30] Huang Y. Hu H. Liu L. Ye J. Wang Z. Que B. (2019). Interleukin-12p35 deficiency reverses the Th1/Th2 imbalance, aggravates the Th17/treg imbalance, and ameliorates atherosclerosis in ApoE−/− mice. Mediat. Inflamm. 2019, 3152040 10.1155/2019/3152040 PMC6481022 31093011

[B31] Hwang S. He Y. Xiang X. Seo W. Kim S. J. Ma J. (2020). Interleukin-22 ameliorates neutrophil-driven nonalcoholic steatohepatitis through multiple targets. Hepatology. 72 (2), 412–429. 10.1002/hep.31031 31705800 PMC7210045

[B32] Khalil M. R. El-Demerdash R. S. Elminshawy H. H. Mehanna E. T. Mesbah N. M. Abo-Elmatty D. M. (2020). Therapeutic effect of bone marrow mesenchymal stem cells in a rat model of carbon tetrachloride induced liver fibrosis. Biomed. J. S2319-4170 (20), 30048–30052. 10.1016/j.bj.2020.04.011 PMC8640564 32389821

[B33] Kim N. G. Kullar R. Khalil H. Saab S. (2020). Meeting the WHO hepatitis C virus elimination goal: review of treatment in paediatrics. J. Viral Hepat. 27 (8), 762–769. 10.1111/jvh.13317 32386099

[B34] Kong B. Liu G. B. Zhang J. A. Fu X. X. Xiang W. Y. Gao Y. C. (2016). Elevated serum IL-35 and increased expression of IL-35-p35 or -EBI3 in CD4(+)CD25(+) T cells in patients with active tuberculosis. Am. J. Transl. Res. 8 (2), 623–633. 27158354 PMC4846911

[B35] Kovalova N. Boyles J. Wen Y. Witcher D. R. Brown-Augsburger P. L. Wroblewski V. J. (2020). Validation of a de-immunization strategy for monoclonal antibodies using cynomolgus macaque as a surrogate for human. Biopharm. Drug Dispos. 41 (3), 111–125. 10.1002/bdd.2222 32080869

[B36] Lan Y. T. Wang Z. L. Tian P. Gong X. N. Fan Y. C. Wang K. (2019). Treg/Th17 imbalance and its clinical significance in patients with hepatitis B-associated liver cirrhosis. Diagn. Pathol. 14 (1), 114 10.1186/s13000-019-0891-4 31639000 PMC6805395

[B37] Li H. Zhang Y. X. Liu Y. Wang Q. (2016). Effect of IL-17 monoclonal antibody Secukinumab combined with IL-35 blockade of Notch signaling pathway on the invasive capability of hepatoma cells. Genet. Mol. Res. 15 (2), 15028174 10.4238/gmr.15028174 27420998

[B38] Li T. Huang Y. Liu P. Liu Y. Guo J. Zhang W. (2018). Lower plasma levels of IL-35 in patients with primary biliary cirrhosis. Tohoku J. Exp. Med. 244 (2), 123–131. 10.1620/tjem.244.123 29445068

[B39] Li Y. Yao L. Liu S. Wu J. Xia L. Shen H. (2019). Correlation between serum IL-35 levels and bone loss in postmenopausal women with rheumatoid arthritis. Mediat. Inflamm. 2019, 9139145 10.1155/2019/9139145 PMC6732582 31534439

[B40] Li W. Gao R. Xin T. Gao P. (2020a). Different expression levels of interleukin-35 in asthma phenotypes. Respir. Res. 21 (1), 89 10.1186/s12931-020-01356-6 32295589 PMC7160921

[B41] Li X. Zhong T. Tang R. Wu C. Xie Y. Liu F. (2020b). PD-1 and PD-L1 expression in peripheral CD4/CD8+ T cells is restored in the partial remission phase in type 1 diabetes. J. Clin. Endocrinol. Metab. 105 (6), dgaa130 10.1210/clinem/dgaa130 32236416

[B42] Li Y. Yin S. Chen Y. Zhang Q. Huang R. Jia B. (2020c). Hepatitis B virus-induced hyperactivation of B cells in chronic hepatitis B patients via TLR4. J. Cell Mol. Med. 24 (11), 6096–6106. 10.1111/jcmm.15202 32391647 PMC7294113

[B43] Lian M. Zhang J. Zhao L. Chen X. Peng Y. Wang Q. (2019). Interleukin-35 regulates immune microenvironment of autoimmune hepatitis through inducing the expansion of myeloid-derived suppressor cells. Front. Immunol. 10, 2577 10.3389/fimmu.2019.02577 31787974 PMC6854006

[B44] Lim T. S. Lee H. W. Lee J. I. Kim I. H. Lee C. H. Jang B. K. (2020). Predictive score for hepatocellular carcinoma after hepatitis B e antigen loss in patients treated with entecavir or tenofovir. J. Viral Hepat. 27 (10), 1052–1060. 10.1111/jvh.13316 32383246

[B45] Liu S. Zhang Q. Shao X. Wang W. Zhang C. Jin Z. (2017). An immunosuppressive function of interleukin-35 in chronic hepatitis C virus infection. Int. Immunopharm. 50, 87–94. 10.1016/j.intimp.2017.06.015 28644966

[B46] Liwinski T. Casar C. Ruehlemann M. C. Bang C. Sebode M. Hohenester S. (2020). A disease-specific decline of the relative abundance of Bifidobacterium in patients with autoimmune hepatitis. Aliment. Pharmacol. Ther. 51 (12), 1417–1428. 10.1111/apt.15754 32383181

[B47] Long J. Zhang X. Wen M. Kong Q. Lv Z. An Y. (2013). IL-35 over-expression increases apoptosis sensitivity and suppresses cell growth in human cancer cells. Biochem. Biophys. Res. Commun. 430 (1), 364–369. 10.1016/j.bbrc.2012.11.004 23154182

[B48] Long J. Guo H. Cui S. Zhang H. Liu X. Li D. (2016). IL-35 expression in hepatocellular carcinoma cells is associated with tumor progression. Oncotarget. 7 (29), 45678–45686. 10.18632/oncotarget.10141 27329841 PMC5216752

[B49] Luo M. Peng H. Chen P. Zhou Y. (2019). The immunomodulatory role of interleukin-35 in fibrotic diseases. Expet Rev. Clin. Immunol. 15 (4), 431–439. 10.1080/1744666X.2019.1564041 30590954

[B50] Ma Y. Liu X. Wei Z. Wang X. Xu D. Dai S. (2014). The expression of a novel anti-inflammatory cytokine IL-35 and its possible significance in childhood asthma. Immunol. Lett. 162 (1 Pt A), 11–17. 10.1016/j.imlet.2014.06.002 24970690

[B51] Martinello M. Bajis S. Dore G. J. (2020). Progress toward hepatitis C virus elimination: therapy and implementation. Gastroenterol. Clin. N. Am. 49 (2), 253–277. 10.1016/j.gtc.2020.01.005 32389362

[B52] Mendez-Sanchez N. Valencia-Rodriguez A. Coronel-Castillo C. Vera-Barajas A. Contreras-Carmona J. Ponciano-Rodriguez G. (2020). The cellular pathways of liver fibrosis in non-alcoholic steatohepatitis. Ann. Transl. Med. 8 (6), 400 10.21037/atm.2020.02.184 32355844 PMC7186641

[B53] Minaga K. Watanabe T. Chung H. Kudo M. (2019). Autoimmune hepatitis and IgG4-related disease. World J. Gastroenterol. 25 (19), 2308–2314. 10.3748/wjg.v25.i19.2308 31148902 PMC6529891

[B54] Ming D. Yu X. Guo R. Deng Y. Li J. Lin C. (2015). Elevated TGF-beta1/IL-31 pathway is associated with the disease severity of hepatitis B virus-related liver cirrhosis. Viral Immunol. 28 (4), 209–216. 10.1089/vim.2014.0142 25710085

[B55] Mohamadi-Zarch S. M. Baluchnejadmojarad T. Nourabadi D. Khanizadeh A. M. Roghani M. (2020). Protective effect of diosgenin on LPS/D-Gal-induced acute liver failure in C57BL/6 mice. Microb. Pathog. 146, 104243 10.1016/j.micpath.2020.104243 32389705

[B56] Peng M. Qiang L. Xu Y. Li C. Li T. Wang J. (2019). IL-35 ameliorates collagen-induced arthritis by promoting TNF-alpha-induced apoptosis of synovial fibroblasts and stimulating M2 macrophages polarization. FEBS J. 286 (10), 1972–1985. 10.1111/febs.14801 30834683

[B57] Petta S. Craxi A. (2020). Extrahepatic manifestations of chronic viral C hepatitis. Gastroenterol. Clin. N. Am. 49 (2), 347–360. 10.1016/j.gtc.2020.01.012 32389367

[B58] Popkova T. Hajek R. Jelinek T. (2020). Monoclonal antibodies in the treatment of AL amyloidosis: co-targetting the plasma cell clone and amyloid deposits. Br. J. Haematol. 189 (2), 228–238. 10.1111/bjh.16436 32072615

[B59] Qiu X. Wang X. Song Y. Chen L. (2016). Plasma level of interleukin-35 as an independent prognostic indicator in hepatocellular carcinoma. Dig. Dis. Sci. 61 (12), 3513–3521. 10.1007/s10620-016-4270-7 27699510

[B60] Roderburg C. Wree A. Demir M. Schmelzle M. Tacke F. (2020). The role of the innate immune system in the development and treatment of hepatocellular carcinoma. Hepat Oncol. 7 (1), HEP17 10.2217/hep-2019-0007 32273975 PMC7137177

[B61] Roth K. Strickland J. Copple B. L. (2020). Regulation of macrophage activation in the liver after acute injury: role of the fibrinolytic system. World J. Gastroenterol. 26 (16), 1879–1887. 10.3748/wjg.v26.i16.1879 32390699 PMC7201151

[B62] Sandusadee N. Sukeepaisarnjaroen W. Suttichaimongkol T. (2020). Prognostic factors for remission, relapse, and treatment complications in type 1 autoimmune hepatitis. Heliyon. 6 (4), e03767 10.1016/j.heliyon.2020.e03767 32382677 PMC7203077

[B63] Schneider S. Kadletz L. Wiebringhaus R. Kenner L. Selzer E. Fureder T. (2018). PD-1 and PD-L1 expression in HNSCC primary cancer and related lymph node metastasis—impact on clinical outcome. Histopathology. 73 (4), 573–584. 10.1111/his.13646 29742291

[B64] Shao X. Wu B. Cheng L. Li F. Zhan Y. Liu C. (2018). Distinct alterations of CD68(+)CD163(+) M2-like macrophages and myeloid-derived suppressor cells in newly diagnosed primary immune thrombocytopenia with or without CR after high-dose dexamethasone treatment. J. Transl. Med. 16 (1), 48 10.1186/s12967-018-1424-8 29499727 PMC5833082

[B65] Shi M. Wei J. Dong J. Meng W. Ma J. Wang T. (2015). Function of interleukin-17 and -35 in the blood of patients with hepatitis B-related liver cirrhosis. Mol. Med. Rep. 11 (1), 121–126. 10.3892/mmr.2014.2681 25323532 PMC4237084

[B66] Shigeoka M. Koma Y. I. Kodama T. Nishio M. Akashi M. Yokozaki H. (2020). Intraepithelial CD163(+) macrophages in tongue leukoplakia biopsy: a promising tool for cancer screening. Oral Dis. 26 (3), 527–536. 10.1111/odi.13269 31886947

[B67] Shouse G. Nikolaenko L. (2019). Targeting the JAK/STAT pathway in T cell lymphoproliferative disorders. Curr. Hematol. Malig. Rep. 14 (6), 570–576. 10.1007/s11899-019-00545-5 31741284

[B68] Skrombolas D. Wylie I. Maharaj S. Frelinger J. G. (2015). Characterization of an IL-12 p40/p35 truncated fusion protein that can inhibit the action of IL-12. J. Interferon Cytokine Res. 35 (9), 690–697. 10.1089/jir.2014.0176 25938719 PMC4560857

[B69] Slawek A. Lorek D. Kedzierska A. E. Chelmonska-Soyta A. (2020). Regulatory B cells with IL-35 and IL-10 expression in a normal and abortion-prone murine pregnancy model. Am. J. Reprod. Immunol. 83 (3), e13217 10.1111/aji.13217 31821644

[B70] Spyrou E. Smith C. I. Ghany M. G. (2020). Hepatitis B: current status of therapy and future therapies. Gastroenterol. Clin. N. Am. 49 (2), 215–238. 10.1016/j.gtc.2020.01.003 PMC7444867 32389360

[B71] Su L. C. Liu X. Y. Huang A. F. Xu W. D. (2018). Emerging role of IL-35 in inflammatory autoimmune diseases. Autoimmun. Rev. 17 (7), 665–673. 10.1016/j.autrev.2018.01.017 29729445

[B72] Su F. Berry K. Ioannou G. N. (2020). No difference in hepatocellular carcinoma risk between chronic hepatitis B patients treated with entecavir versus tenofovir. Gut. [Epub ahead of print]. 10.1136/gutjnl-2019-319867 32229544

[B73] Sullivan J. A. Tomita Y. Jankowska-Gan E. Lema D. A. Arvedson M. P. Nair A. (2020). Treg-cell-derived IL-35-coated extracellular vesicles promote infectious tolerance. Cell Rep. 30 (4), 1039–1051. 10.1016/j.celrep.2019.12.081 31995748 PMC7042971

[B74] Teng D. K. Liu Y. Lv Y. F. Wang L. Zhang W. Wang J. P. (2019). Elevated interleukin-35 suppresses liver inflammation by regulation of T helper 17 cells in acute hepatitis B virus infection. Int. Immunopharm. 70, 252–259. 10.1016/j.intimp.2019.02.048 30851705

[B75] Terayama H. Yoshimoto T. Hirai S. Naito M. Qu N. Hatayama N. (2014). Contribution of IL-12/IL-35 common subunit p35 to maintaining the testicular immune privilege. PLoS One. 9 (4), e96120 10.1371/journal.pone.0096120 24760014 PMC3997559

[B76] Tsai C. L. Chang J. S. Yu M. C. Lee C. H. Chen T. C. Chuang W. Y. (2020). Functional genomics identifies hepatitis-induced STAT3-TYRO3-STAT3 signaling as a potential therapeutic target of hepatoma. Clin. Canc. Res. 26 (5), 1185–1197. 10.1158/1078-0432.CCR-18-3531 31831556

[B77] Tumino N. Casetti R. Fabbri G. Cimini E. Romanelli A. Turchi F. (2017). In HIV/HCV co-infected patients T regulatory and myeloid-derived suppressor cells persist after successful treatment with directly acting antivirals. J. Hepatol. 67 (2), 422–424. 10.1016/j.jhep.2017.03.036 28411041

[B78] Wang Y. Dong J. Meng W. Ma J. Wang N. Wei J. (2014). Effects of phased joint intervention on IL-35 and IL-17 expression levels in patients with portal hypertension. Int. J. Mol. Med. 33 (5), 1131–1139. 10.3892/ijmm.2014.1662 24549402

[B79] Wang J. Tao Q. Wang H. Wang Z. Wu F. Pan Y. (2015). Elevated IL-35 in bone marrow of the patients with acute myeloid leukemia. Hum. Immunol. 76 (9), 681–686. 10.1016/j.humimm.2015.09.020 26431888

[B80] Wang K. Gong H. Chai R. Yuan H. Chen Y. Liu J. (2018a). RETRACTED: aberrant frequency of IL-35 producing B cells in colorectal cancer patients. Cytokine. 102, 206–210. 10.1016/j.cyto.2017.10.011 29054723

[B81] Wang W. Guo H. Li H. Yan Y. Wu C. Wang X. (2018b). Interleukin-35 gene-modified mesenchymal stem cells protect concanavalin A-induced fulminant hepatitis by decreasing the interferon gamma level. Hum. Gene Ther. 29 (2), 234–241. 10.1089/hum.2017.171 29054137

[B82] Wei K. Jiang B. C. Guan J. H. Zhang D. N. Zhang M. X. Wu J. L. (2018). Decreased CD4(+)CD25(+)CD127(dim/-) regulatory T cells and T helper 17 cell responsiveness to toll-like receptor 2 in chronic hepatitis C patients with daclatasvir plus asunaprevir therapy. Viral Immunol. 31 (8), 559–567. 10.1089/vim.2018.0055 30067145

[B83] Wetzel A. Scholtka B. Gerecke C. Kleuser B. (2020). Epigenetic histone modulation contributes to improvements in inflammatory bowel disease via EBI3. Cell. Mol. Life Sci. 77 (23), 5017–5030. 10.1007/s00018-020-03451-9 31955243 PMC7658076

[B84] Xing H. Tian G. (2020). Increased Interleukin-35 suppresses peripheral CD14(+) monocytes function in patients with Kawasaki disease. BMC Immunol. 21 (1), 17 10.1186/s12865-020-00348-x 32276581 PMC7149926

[B85] Xu W. Yang Y. Hu Z. Head M. Mangold K. A. Sullivan M. (2020a). LyP-1-Modified oncolytic adenoviruses targeting transforming growth factor beta inhibit tumor growth and metastases and augment immune checkpoint inhibitor therapy in breast cancer mouse models. Hum. Gene Ther. 31 (15–16), 863–880. 10.1089/hum.2020.078 32394753 PMC7462024

[B86] Xu X. Wang R. Wu R. Yan W. Shi T. Jiang Q. (2020b). Trehalose reduces bone loss in experimental biliary cirrhosis rats via ERK phosphorylation regulation by enhancing autophagosome formation. Faseb. J. 34 (6), 8402–8415. 10.1096/fj.201902528RRR 32367591

[B87] Yang L. Jia S. Shao X. Liu S. Zhang Q. Song J. (2019a). Interleukin-35 modulates the balance between viral specific CD4(+)CD25(+)CD127(dim/-) regulatory T cells and T helper 17 cells in chronic hepatitis B virus infection. Virol. J. 16 (1), 48 10.1186/s12985-019-1158-0 30992023 PMC6469219

[B88] Yang L. Shao X. Jia S. Zhang Q. Jin Z. (2019b). Interleukin-35 dampens CD8(+) T cells activity in patients with non-viral hepatitis-related hepatocellular carcinoma. Front. Immunol. 10, 1032 10.3389/fimmu.2019.01032 31134088 PMC6514160

[B89] Yang J. Ma C. Zhao Y. Fan A. Zou X. Pan Z. (2020a). Hepatitis B virus core particles containing a conserved region of the G protein combined with interleukin-35 protected mice against respiratory syncytial virus infection without vaccine-enhanced immunopathology. J. Virol. 94 (13), e00007–e00020. 10.1128/JVI.00007-20 32321805 PMC7307162

[B90] Yang L. Zhang Q. Song J. Wang W. Jin Z. (2020b). Interleukin-35 suppresses CD8(+) T cell activity in patients with viral hepatitis-induced acute-on-chronic liver failure. Dig. Dis. Sci. 65 (12), 3614–3623. 10.1007/s10620-020-06077-w 31974915

[B91] Ye Y. Xie X. Yu J. Zhou L. Xie H. Jiang G. (2010). Involvement of Th17 and Th1 effector responses in patients with hepatitis B. J. Clin. Immunol. 30 (4), 546–555. 10.1007/s10875-010-9416-3 20393789

[B92] Ye Z. Jiang Y. Sun D. Zhong W. Zhao L. Jiang Z. (2019). The plasma interleukin (IL)-35 level and frequency of circulating IL-35(+) regulatory B cells are decreased in a cohort of Chinese patients with new-onset systemic lupus erythematosus. Sci. Rep. 9 (1), 13210 10.1038/s41598-019-49748-z 31519970 PMC6744462

[B93] Yoshimura K. Suzuki Y. Inoue Y. Tsuchiya K. Karayama M. Iwashita Y. (2020). CD200 and CD200R1 are differentially expressed and have differential prognostic roles in non-small cell lung cancer. OncoImmunology. 9 (1), 1746554 10.1080/2162402X.2020.1746554 32395395 PMC7204521

[B94] Zhang J. Lin Y. Li C. Zhang X. Cheng L. Dai L. (2016). IL-35 decelerates the inflammatory process by regulating inflammatory cytokine secretion and M1/M2 macrophage ratio in psoriasis. J. Immunol. 197 (6), 2131–2144. 10.4049/jimmunol.1600446 27527600

[B95] Zhang M. X. Gan W. Jing C. Y. Zheng S. S. Zhang J. Shen H. J. (2018). Overexpression of interleukin-35 in intrahepatic cholangiocarcinoma is a prognostic indicator after curative resection. Canc. Sci. 109 (4), 1195–1206. 10.1111/cas.13535 PMC5891208 29446854

[B96] Zhang J. Zhang Y. Wang Q. Li C. Deng H. Si C. (2019). Interleukin-35 in immune-related diseases: protection or destruction. Immunology. 157 (1), 13–20. 10.1111/imm.13044 30681737 PMC6459776

[B97] Zhang J. Lian M. Li B. Gao L. Tanaka T. You Z. (2020). Interleukin-35 promotes Th9 cell differentiation in IgG4-related disorders: experimental data and review of the literature. Clin. Rev. Allergy Immunol. [Epub ahead of print]. 10.1007/s12016-020-08803-8 32712804

[B98] Zhao N. Hao J. Ni Y. Luo W. Liang R. Cao G. (2011). Vgamma4 gammadelta T cell-derived IL-17A negatively regulates NKT cell function in Con A-induced fulminant hepatitis. J. Immunol. 187 (10), 5007–5014. 10.4049/jimmunol.1101315 21987663

[B99] Zheng X. F. Hu X. Y. Ma B. Fang H. Zhang F. Mao Y. F. (2018). Interleukin-35 attenuates D-galactosamine/lipopolysaccharide-induced liver injury via enhancing interleukin-10 production in kupffer cells. Front. Pharmacol. 9, 959 10.3389/fphar.2018.00959 30197594 PMC6117388

[B100] Zhou X. Xia N. Lv B. Tang T. Nie S. Zhang M. (2020). Interleukin 35 ameliorates myocardial ischemia-reperfusion injury by activating the gp130-STAT3 axis. Faseb. J. 34 (2), 3224–3238. 10.1096/fj.201901718RR 31917470

